# Increased synovial immunohistochemistry reactivity of TGF-β1 in erosive peripheral psoriatic arthritis

**DOI:** 10.1186/s12891-023-06339-4

**Published:** 2023-03-30

**Authors:** Jose A. Pinto Tasende, M. Fernandez-Moreno, M. E. Vazquez-Mosquera, J. C. Fernandez-Lopez, N. Oreiro-Villar, F. J. De Toro Santos, F. J. Blanco-García

**Affiliations:** 1 Department of Rheumatology-INIBIC, Complexo Hospitalario Universitario de A Coruña, 84 Xubias de Arriba Road, 15006, A Coruña, Spain; 2 INIBIC, Complexo Hospitalario Universitario de A Coruña, A Coruña, Spain; 3 Department of Rheumatology, Complexo Hospitalario Universitario de A Coruña, Universidade de A Coruña, A Coruña, Spain; 4 Department of Rheumatology-INIBIC, Complexo Hospitalario Universitario de A Coruña, Universidade de A Coruña, A Coruña, Spain

**Keywords:** TGF-β1, IHC reactivity, mRNA expression, Psoriatic arthritis, Synovial membrane

## Abstract

**Background:**

Immune and non-immune cells contribute to the pathology of chronic arthritis, and they can contribute to tissue remodeling and repair as well as disease pathogenesis. The present research aimed to analyze inflammation and bone destruction/regeneration biomarkers in patients with psoriatic arthritis (PsA), rheumatoid arthritis (RA), osteoarthritis (OA), and ankylosing spondylitis (AS).

**Methods:**

Samples were obtained from the inflamed knee of patients with knee arthritis who had been referred for undergoing arthroscopies. The synovial membrane was processed for pathological description, IHC analysis, and quantification of mRNA expression ratio by qRT-PCR. Serum levels of TGF-β1, IL-23, IL-6, IL-17 A, IL-22, Dkk1, Sclerostin, BMP2, BMP4, Wnt1, and Wnt5a were measured by ELISA. All these data were analyzed and compared with the demographic, clinical, blood tests, and radiological characteristics of the patients.

**Results:**

The synovial membrane samples were obtained from 42 patients for IHC, extraction, and purification of RNA for synovial mRNA expression analysis, and serum for measuring protein levels from 38 patients. IHC reactivity for TGF-β1 in the synovial tissue was higher in patients with psoriatic arthritis (p 0.036) and was positively correlated with IL-17 A (r = 0.389, p = 0.012), and Dkk1 (r = 0.388, p = 0.012). Gene expression of the IL-17 A was higher in PsA patients (p = 0.018) and was positively correlated with Dkk1 (r = 0.424, p = 0.022) and negatively correlated with BMP2 (r = -0.396, p = 0.033) and BMP4 (r = -0.472, p = 0.010). It was observed that IHC reactivity for TGF-β1 was higher in patients with erosive PsA (p = 0.024).

**Conclusions:**

The IHC reactivity of TGF-β1 in synovial tissue was higher in patients with erosive psoriatic arthritis, and TGF-β1 was in relation to higher levels of gene expression of IL-17 A and Dkk1.

## Introduction

Psoriatic arthritis (PsA) belongs to the spondylarthritis (SpA) group, which encompasses a group of diseases sharing genetic, pathophysiological, clinical, and radiological features, where bone remodeling alterations appear when there is an imbalance between bone resorption and bone formation. Osteolysis in patients with rheumatoid arthritis (RA) results from an imbalance in which bone resorption by osteoclasts is favored over bone formation by osteoblasts,with the inflammatory cells infiltrating the synovial tissues, resulting in synovial hyperplasia, angiogenesis, cartilage destruction, and bone erosion in the diarthrodial joints, where local and systemic factors interrupt the physiological bone remodeling process [1]. Depending on the local microenvironment, inflammation has very different effects on bone, inducing bone loss in the joints and periarticular bone or bone formation in the enthesis areas in patients with axial and peripheral SpA [2]. In the last years, a great deal has been learned about the differentiation and function of osteoclasts [3], and now it is appreciated that osteoblast-mediated bone formation is also inhibited in the rheumatoid joint, limiting erosion repair [1], while osteoblast function is increased for producing new bone in SpA [4].

The physio-pathological differences between RA and SpA are rooted in the interaction network of proinflammatory cytokines and are probably related to the different expression of IL-17 A and TNFα, two cytokines that strongly promote osteoclastogenesis and the development of focal bone erosions [5]. TNFα is the main proinflammatory cytokine in RA and promotes bone erosion by triggering osteoclast on through the RANK-RANKL system and by suppressing osteoblast bone formation through the overexpression of Dickkopf-1 (Dkk1), a potent inhibitor of the anabolic Wnt bone signaling pathway [5, 6]. In RA, where the overexpression of TNFα is higher than IL-17 A, bone resorption prevails over bone formation. Unlike TNFα, IL-17 A also promotes osteogenesis, particularly at inflamed sites that experience mechanical stress, as is the case with entheses in animal models of SpA, [7] where fibroblast-like synoviocytes exposed to IL-17 A differentiate into osteoblasts.

Bone morphogenetic protein (BMP) signaling could have an anti-inflammatory role in the control and maintenance of low levels of pro-inflammatory factors in healthy joints or in the early stages of RA. It is a critical pathway for osteoblast differentiation and function and this pathway plays a role in bone formation in SpA, with higher BMP2 and 4 serum levels in the serum of ankylosing spondylitis (AS) patients and having a significant correlation with spinal radiograph scores by developing spinal fusion [8].

PsA is an immune-mediated chronic inflammatory arthritis, where skin-resident cells such as keratinocytes, γδT cells, and innate lymphoid cells express IL-17 A, which also inhibits osteoblasts and osteocytes function through Wnt signaling [9] and IL-17 A is a bone-destroying cytokine that is involved in immune-mediated bone diseases and can exert a negative effect on bone by promoting osteoclastogenesis and initiates an immunologic cascade that is associated with synovial inflammation, bone destruction, and juxta-articular new bone formation [10]. Furthermore, the clinical trials of IL-17 A inhibitors in PsA have shown improvement in the signs and symptoms of active PsA [11], and they can inhibit the progression of bone erosion and maintain bone stability [12]. Therefore, we hypothesize the synovial expression of TGF-β1 is a differentiating factor for the development of erosive peripheral disease in psoriatic arthritis (TGF-β1 can prime IL-17 A), and synovial expression of Dkk1 may be related to peripheral joint destruction as in rheumatoid arthritis.

The present study aimed to analyze proteins of inflammation and bone destruction and regeneration in serum, gene expression, and immunohistochemistry (IHC) reactivity in the synovial membrane in patients with PsA, compared with rheumatoid arthritis, osteoarthritis, and AS.

## Materials and methods

### Patients and samples

This is a descriptive cross-sectional study in patients fulfilling the CASPAR criteria for psoriatic arthritis (n = 15) to determine the expression of the biomarkers that may be associated with joint inflammation (TGF-β1, IL-23, IL-6, IL-17 A, and IL-22) and with destruction or new bone formation (DKK1, Sclerostin, BMP2, BMP4, Wnt1, and Wnt5a) using immunohistochemistry and qRT-PCR in synovial tissue and by measuring the synovial fluid and serum levels. The expression of these proteins in patients with PsA was compared to their expression in synovial tissue samples from the knee joint and serum from patients with RA (n = 8), OA (n = 18), and AS (n = 4). The patients underwent knee arthroscopy between 2009 and 2013 because they clinically presented with knee joint swelling and tenderness that did not improve with the medical treatment indicated for their diagnosed disease. In December 2018, it was verified that the patient’s clinical diagnosis had not changed. Those patients whose macroscopic or microscopic characteristics of the synovial membrane samples presented characteristics different from the underlying pathology (e.g., deposits of urate or PPCD crystals, pigmented villonodular synovitis or vasculitis) that could interfere with the determinations made were excluded, as well as poorly labeled or unlocatable samples.

Patients were evaluated every 3 to 4 months, and the treatment was changed if the disease remained active (≥ 2 swollen and tender joints). Therapy with methotrexate was initiated (up to 20 mg/week if tolerated), and if no response occurred or adverse events were noted, patients were switched to anti-TNF-a or to combined therapy, according to their rheumatologist’s judgment. Clinical and biological data (tender/swollen joint counts 66/68, C-reactive protein (CRP) and erythrocyte sedimentation rate (ESR), disease-modifying antirheumatic drugs (DMARDs), and biologic therapy administered) were collected during the study inclusion and the last clinical control. None of the patients was being treated with biologics, and eight of the patients with PsA were on treatment with methotrexate (MTX).

The Kellgren-Lawrence (KL) scale was used to establish the degree of osteoarthritis in the knee [13]. The evaluation of axial radiological damage was also performed using sacroiliac X-ray and through the classification of sacroiliitis according to the modified New York criteria [14] as well as to the presence/absence of erosive peripheral joint damage.

This study was carried out at the A Coruña Biomedical Research Institute (INIBIC). The study was approved by the Clinical Research Ethics Committee (CEIC), under number 2011/301, and performed according to the Declaration of Helsinki. All study patients provided written informed consent.

### Arthroscopies

At first, peripheral blood was extracted from a forearm vein. Synovial tissue samples were obtained surgically from the knee joint by means of arthroscopy under local anesthesia.

Arthroscopy was performed under diagnostic and/or therapeutic (lavage) conditions with a 2.7-mm arthroscope (Storz, Tullingen, Germany). Eight synovial tissue samples were obtained from the suprapatellar pouch and the medial and lateral gutter in each patient. Four samples were fixed in 4% formaldehyde and embedded in paraffin wax for immunohistochemistry, and the remaining four were collected on RLT lysis buffer (Qiagen, Crawley, West Sussex, UK) for RNA extraction [15].

Arthroscopic joint lavage is a formal joint lavage that takes place in addition to a visual inspection of the structures of the knee joint at the same time [16]. The amount of irrigation fluid (saline serum 0.9% at 5ºC) used was 5000 mL, and the procedure took place over 30 min.

The biopsies from each patient were collected, carved, and fixed in the operating room and, together with the blood samples, transferred to the INIBIC facilities in the shortest possible time (less than 1 h). Once in the laboratory, the samples were registered in the data bank (Biobank) using NorayBanks software to guarantee the confidentiality of the procedure. After encoding, they were processed according to the techniques described below.

### Histopathological analysis and immunohistochemistry: quantification of protein expression in IHC staining

The synovial biopsies from each patient were fixed in the operating room, some were immersed in 4% formaldehyde for up to 24 h, and others were immersed in OCT (cryoprotective medium). They were transported using post-physical fixation in dry ice for storage following an established order in a -80ºC chest in the Basic Research Laboratory at the INIBIC.

Once the inclusion was finished, the sections were stained with hematoxylin-eosin and Masson’s trichrome (H-E, MM-classical histological stains) for the first morphological study. The histopathological analysis was carried out by a pathologist other than the immunohistochemistry, who made a detailed description of it, without images attached to the report.

Indirect immunohistochemical techniques (with paraffin peroxidase) were used in all the biopsies, in which the mouse monoclonal anti-BMP2 ab6285 antibody (clone 65529.111) of Abcam® and rabbit monoclonal anti-Dkk1 antibody were used as the primary antibodies. ab109416 (clone EPR4759) from Abcam®, rabbit anti-BMP2 monoclonal antibody ab 124,715 (clone EPR6211) from Abcam®, mouse monoclonal anti-Wnt5a ab86720 (clone 3D10) from Abcam®, mouse monoclonal antibody anti-TGF-β1 ab64715 (clone 2Ar2) from Abcam®, Abcam® ab79056 rabbit anti-IL-17 A polyclonal antibody, and Dako® K-5007 antibody (EnVision ™ Detection Systems Peroxidase / DAB), were used as the secondary antibodies.

The samples were pretreated with tris-EDTA at pH 9 in Retriever (0.1 M sodium citrate pH 6.1 for Wnt5a), and the positive controls were BMP2 for human small bowel tissue (1: 5000), BMP4 for human colon tissue (1: 1000), Dkk1 for human placenta tissue (1: 1000), Wnt5a for human thyroid tissue (1: 1000), TGF-β1 for human articular cartilage tissue and osteoarthritis (1:50), and IL-17 A for human lymphatic node (1: 1000), and the negatives of the technique were conducted without the use of a primary antibody.

The chromophore that was used includes the chromogen diaminobenzidine (DAB, brown color) and the peroxidase substrate (H2O2). The samples were counterstained with Gill III’s hematoxylin-eosin, dehydrated, and rinsed with xylene. DPX (acrylic resin) was used as a coverslip mounting medium. Image capture was performed with the Olympus BX61 microscope, and the analysis and quantification of the samples were conducted with the Nikon Eclipse microscope, using NIS Elements imaging software.

### Determination of serum levels of proteins: enzyme-linked ImmunoSorbent Assay (ELISA)

In this study, the double sandwich ELISA (DAS) was performed in triplicate, in 96-well microplates. The expression of BMP2, BMP4, Dkk1, Wnt1, Wnt5a, sclerostin, TGF- β1, IL-6, IL-17 A, and IL-22 in the serum was quantified according to the manufacturer’s specifications of each ELISA KIT.

The absorbance was measured in a microplate reader at 450 nm, with the results being extrapolated to the standard curve in each case.

### Determination of gene expression in synovial tissue: quantitative real-time PCR (qRT-PCR)

From the biopsies obtained during the arthroscopies, we performed RNA extraction with TRIzol®Reagent (Life Technologies) following the manufacturer’s instructions. The genetic material obtained was quantified by spectrophotometry with NanoDrop ™ (Thermo Scientific).

Using the Superscript® VILO ™ cDNA synthesis kit (Invitrogen), it was performed the reverse transcription of the RNA. The cDNA that was obtained was amplified by qRT-PCR with LightCycler 480 II equipment (Roche) using the Taqman probes (Light-Cycler® 480 Probes Master (Roche). The 60 S ribosomal protein L13a (RPL13a) was used to normalize the results that were obtained. The qRT-PCR results were analyzed with the qBase plus software (Biogazelle).

### Statistical analysis

The results were expressed as a percentage or as the median and interquartile range (IQR) and categoric variables as frequencies and percentages. Comparisons of qualitative variables were made using the chi-square test and Fisher’s exact test if applicable. The Wilcoxon rank sum test or the Kruskal-Wallis’s test were used to compare the distribution of the numeric variables between the groups. The correlation between the numeric variables was expressed by the Spearman correlation coefficient, and the null hypothesis was tested (coefficient = zero). The correlation between two categoric variables or between one numeric and one categoric variable was assessed by using Fisher´s exact test and the Wilcoxon rank sum test or the Kruskal-Wallis’s test. Univariate and multivariate logistic regression models were performed to evaluate the association of the proteins related to gene expression with demographics, clinical, radiological, and therapeutic features.

The data were statistically analyzed with the SPSS version 21 program (IBM SPSS Statistics). Values of p < 0.05 were considered statistically significant.

## Results

Women were more prevalent in the OA group (p = 0.036), and these patients were older (< 0.0001). Disease duration was higher in the OA group and in the AS group and was associated with a longer period of disease evolution while for the PsA and RA groups was about 3 years (p 0.002). The peripheral joint disease for all groups was mono-articular (knee joint target), except for PsA which had an oligo-articular joint count (0.033). CRP was higher in the AS group, and this was the only group of patients with radiologic sacroiliac damage and positive HLA-B27. Out of 15 PsA patients, only four had erosions on X-ray films, and eight patients were treated with MTX (Table 1).

**Table 1:** Characteristics of patients according to the diseases

All n = 45*	PsA n = 15	RA n = 8	OA n = 18	AS n = 4	p**	
Male, n (%)	10 (66.7)	4 (50.0)	7 (30.9)	4 (100)	0.036	
Age, years	48.0 (40.5–55.5)	42.5 (32.2–58.7)	69.0 (64.5–74.7)	57.5 (49.0-65.2)	< 0.0001	
Disease duration, years	3.0 (1.0–9.0)	2.0 (1.0-5.7)	12.5 (6.0-18.5)	19.5 (8.0-31.7)	0.002	
Tender joints count	2 (1–3)	1 (1–2)	1 (1–1)	1 (1-1.7)	0.033	
Swollen joints count	1 (1–2)	1 (1-1.7)	1 (1–1)	1 (1–1)	0.236	
ESR, mm/1st h	20 (8–36)	15 (7–29)	22 (14–33)	45 (23–69)	0.181	
CRP, mg/dL	0.70 (0.29–1.74)	0.36 (0.21–0.61)	0.44 (0.25–0.53)	2.20 (0.52–3.80)	0.05	
Uricemia, mg/dL	4.8 (4.1–6.3)	5.7 (4.2–7.7)	5.5 (4.5-6.0)	5.2 (4.2–6.9)	0.715	
RF and/or ACPA +, n (%)	0	4 (50)	0	0	0.001	
X-ray Erosions+, n (%)	4 (26.7)	0	0	0	0.032	
X-ray Sacroiliitis+, n (%)	0	0	0	4 (100%)	0.001	
Methotrexate, n (%)	8 (53.3)	0	0	0	0.004	

*Data as median (Q1-Q3) and percentages. ** Chi-square/Fisher exact test) and Kruskal-Wallis test.

RF: rheumatoid factor; ACPA: anti-citrullinated protein antibody

HLA-B27 +: 4 AS

From 45 patients, histopathological analysis of the synovial membrane of PsA patients showed an increase in vessel density in PsA compared with RA, OA, or AS (p 0.035). Other features had not shown differences among patients (Table 2). No statistically significant differences were observed regarding hyperplasia of the synovial lining, villous papillae, lymphoplasmacytic accumulations, lymphocytic or plasmacytoid infiltrates, and fibrosis.

**Table 2:** Synovial histopathology pattern according to the diseases

All n = 45	PsA n = 15	RA n = 8	OA n = 18	AS n = 4	p*	
**Hyperplasia of the Synovial Lining**, n (%)	12 (80)	6 (75)	12 (66.7)	3 (75.0)	0.307	
**Villous papillae**, n (%)	3 (20)	1 (12.5)	3 (16.7)	0 (0)	0.794	
**Lymphoplasmacytoid accumulations**, n (%)	4 (26.7)	1 (12.5)	2 (11.1)	0 (0)	0.453	
**Lymphocytic infiltrate**, n (%)	7 (46.7)	5 (62.5)	6 (33.3)	2 (50.0)	0.183	
**Plasmacytoid infiltrate**, n (%)	4 (26.7)	3 (37.5)	4 (22.2)	2 (50.0)	0.498	
**High vascularity**, n (%)	10 (66.7)	4 (50)	4 (22.2)	0 (0)	0.035	
**Fibrosis**, n (%)	3 (20)	0 (0)	4 (22.2)	1 (25.0)	0.426	

* Chi-square/Fisher exact test).

For the histopathological study, 45 valid samples of the synovial membrane were available, but only from 42 patients for IHC, extraction, and purification of RNA for synovial mRNA expression analysis. Blood was obtained to measure the protein levels in the serum, and only valid samples were available from 38 patients.

**Fig. 1:**
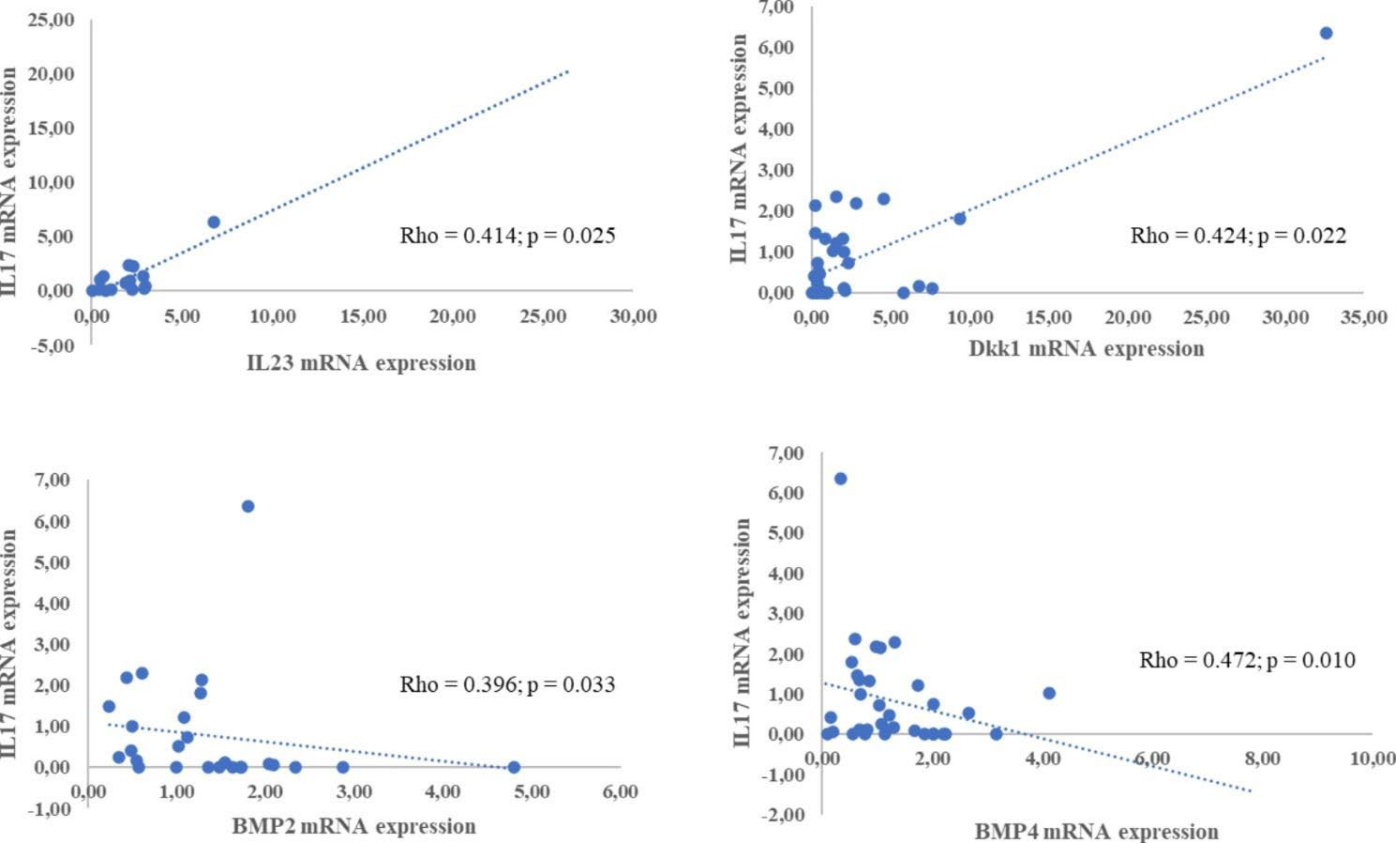
Correlation between gene expression of IL-17 A with IL-23, Dkk1, BMP2, and BMP4

The IL-17 A gene expression in the synovial membrane (Table 3) was higher in PsA patients (p = 0.018) and was positively correlated with IL-23 (p = 0.025), and Dkk1 (p = 0.022), and negatively with BMP2 (p = 0.033), and BMP4 (p = 0.01) (Fig. 1).

**Table 3:** Expression ratios of mRNA in the synovial membrane

mRNA*	All n= 42	PsA n = 15	RA n = 8	OA n = 15	AS n = 4	p
**DKK1**	0.74 (0.33–2.52)	1.22 (0.36–6.41)	0.50 (0.37–2.19)	0.45 (0.20–0.84)	4.32 (0.97–12.23)	0.112
**Sclerostin**	0.0 (0.0-0.69)	0.0 (0.0-0.16)	0.58 (0.0-0.88)	0.0 (0.0-1.77)	0.0 (0.0-0.23)	0.504
**BMP2**	1.28 (0.59–1.72)	1.27 (0.73–1.43)	1.02 (0.52–1.54)	1.68 (1.43–2.28)	1.08 (0.37–1.78)	0.145
**BMP4**	1.07 (0.73–1.84)	1.17 (0.73–1.83)	0.90 (0.73–1.37)	2.01 (0.63–2.29)	1.06 (0.39–1.28)	0.339
**Wnt1**	0.44 (0.24–2.25)	1.44 (0.40–7.95)	0.30 (0.17–2.99)	0.27 (0.0-1.78)	0.60 (0.22–1.94)	0.191
**Wnt5a**	0.62 (0.40–1.25)	0.48 (0.33–1.43)	0.55 (0.47–1.10)	1.15 (0.48–2.30)	0.56 (0.35–0.81)	0.533
**TGFβ1**	0.99 (0.73–1.27)	0.92 (0.61–1.24)	0.99 (0.78–1.54)	1.01 (0.85–1.32)	1.05 (0.68–1.18)	0.921
**IL6**	0.90 (0.42–2.99)	1.85 (0.55–3.34)	0.73 (0.19–2.35)	0.58 (0.38–2.67)	1.74 (0.88–21.48)	0.371
**IL17A**	0.11 (0.0-1.34)	1.64 (0.30–2.25)	0.41 (0.10–0.70)	0.11 (0.00-0.05)	1.09 (0.00-5.31)	**0.018**
**IL22**	0.67 (0.0-1.79)	0.69 (0.12–2.10)	0.73 (0.10–2.20)	0.23 (0.00-0.71)	1.61 (0.49–5.55)	0.342
**IL23**	1.09 (0.41–2.27)	1.16 (0.68–2.33)	0.75 (0.32–1.91)	1.41 (0.54–2.25)	2.94 (2.41–4.85)	0.215

*Data as median (Q1-Q3). Kruskal-Wallis test.

**Fig. 2:**
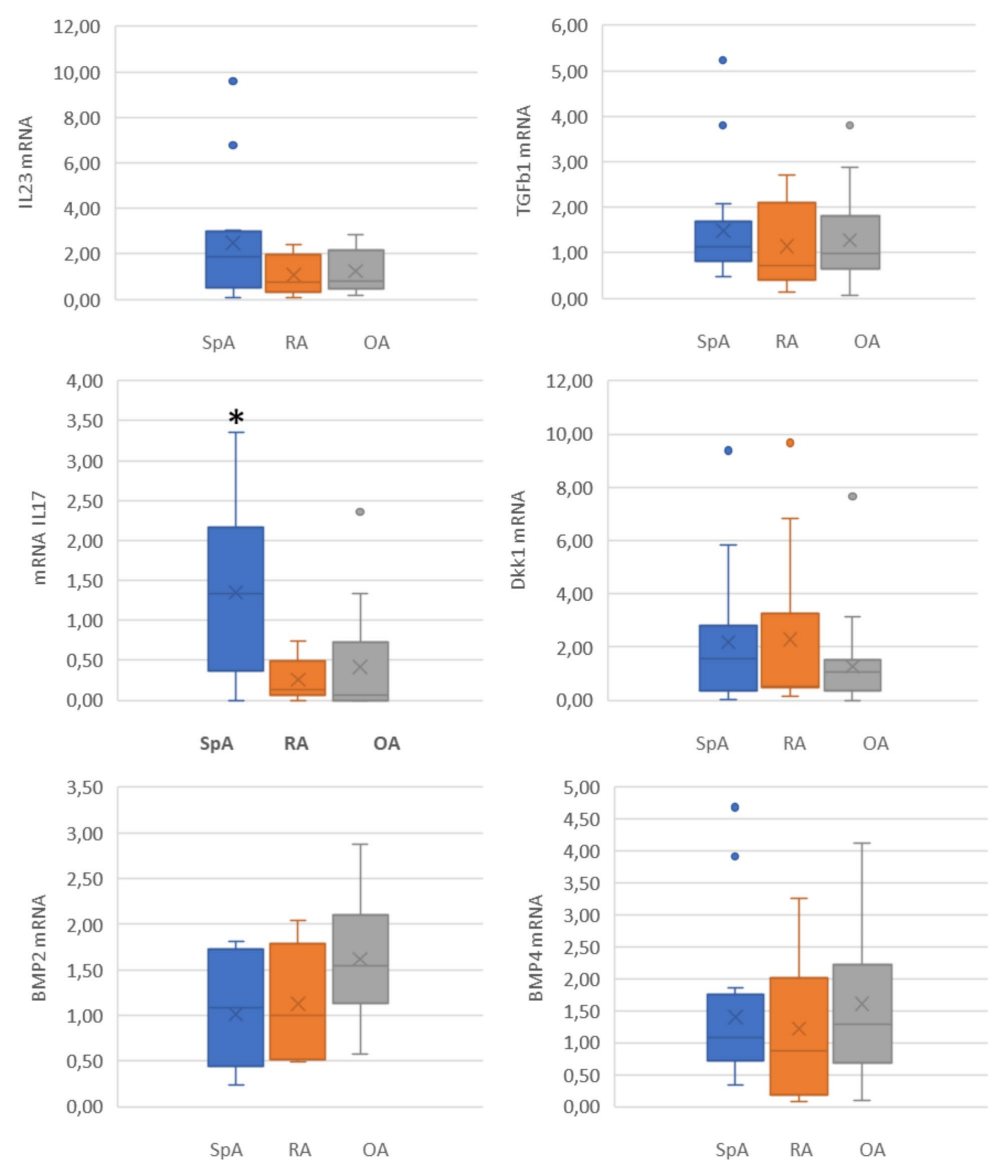
Differences between gene expression of IL-17 A, IL-23, Dkk1, TGF-β1, BMP2, and BMP4 SpA: spondyloarthritis (n= 18): RA: rheumatoid arthritis (n=8); OA: osteoarthritis (n=16) (*) p= 0.001

**Fig. 3:**
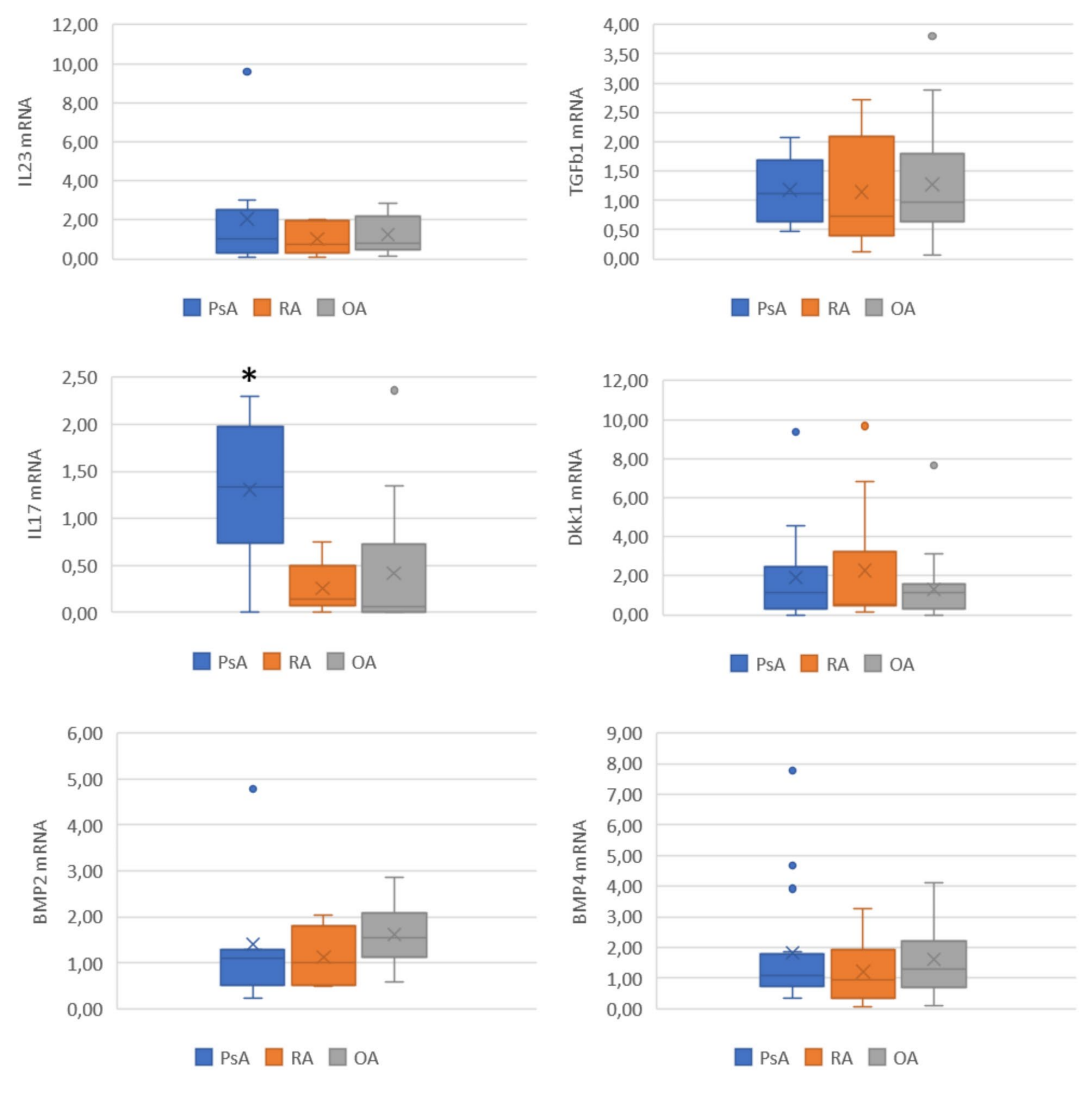
Differences between gene expression of IL-17 A, IL-23, Dkk1, TGF-β1, BMP2, and BMP4 (excluding ankylosing spondylitis patients) PsA: psoriatic arthritis (n= 14); RA: rheumatoid arthritis (n=8); OA: osteoarthritis (n=16) (*) p= 0.018

Patients with diagnoses of peripheral SpA (PsA and AS with knee arthritis) had a higher mRNA expression ratio of IL17A than patients with RA or OA (p = 0.001), and of IL-23, but this last was not statistically significant (Fig. 2). Considering spondyloarthritis patients as a group with shared pathophysiological characteristics, both had higher IL17A gene expression. Separately, patients with AS had higher synovial IL17A gene expression than in OA or RA (p 0.01). However, these data were even higher for PsA patients (p 0.009), as can be seen in Fig. 3. There were no differences between PsA and AS regarding IL-23. When was considered the presence of treatment with MTX, lower levels of IL-17 A gene expression were not observed in patients being treated with MTX (p = 0.703).

**Fig. 4:**
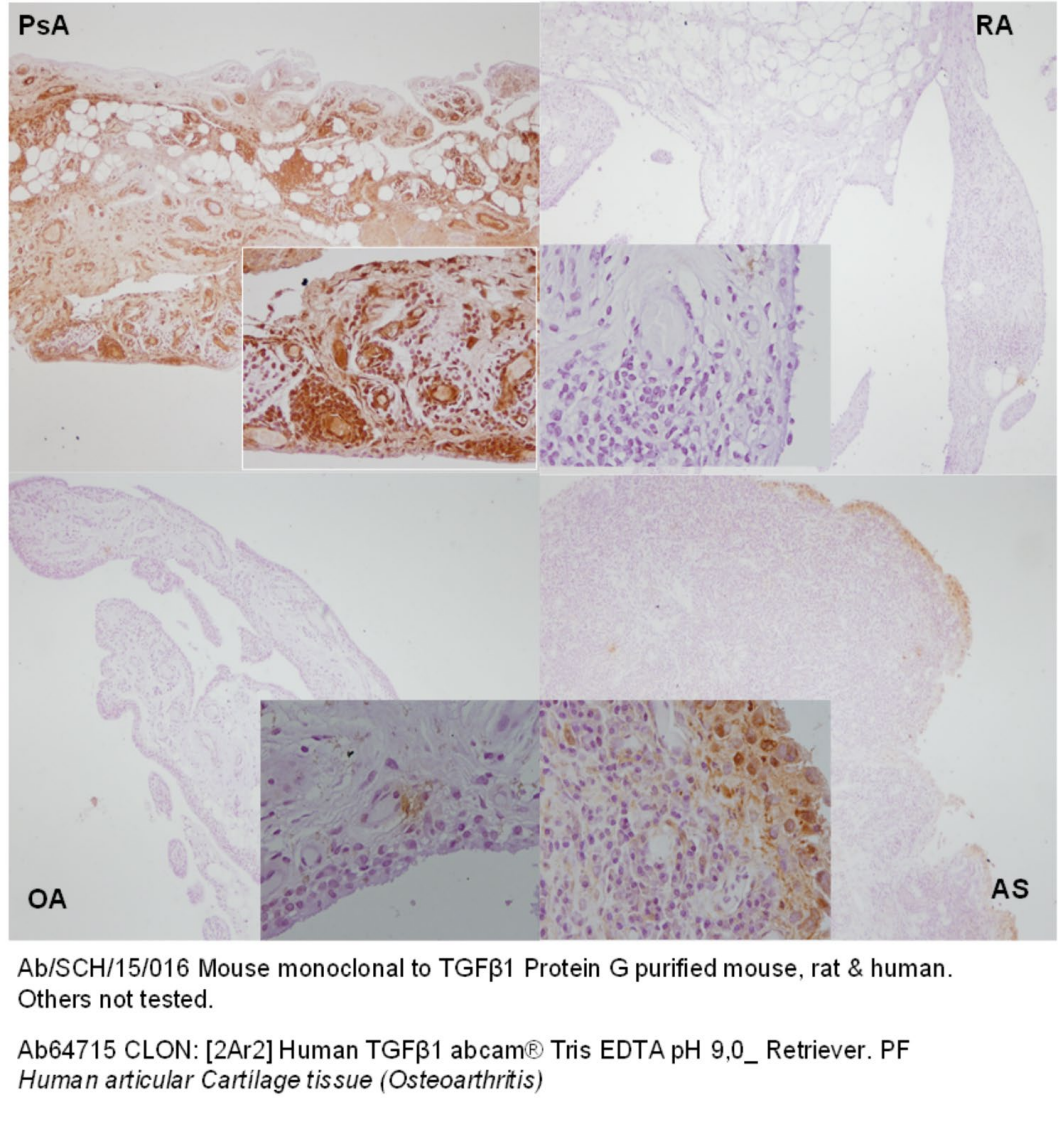
IHC in the synovial membrane of TGFβ1 (brown color) in the four groups

**Fig. 5:**
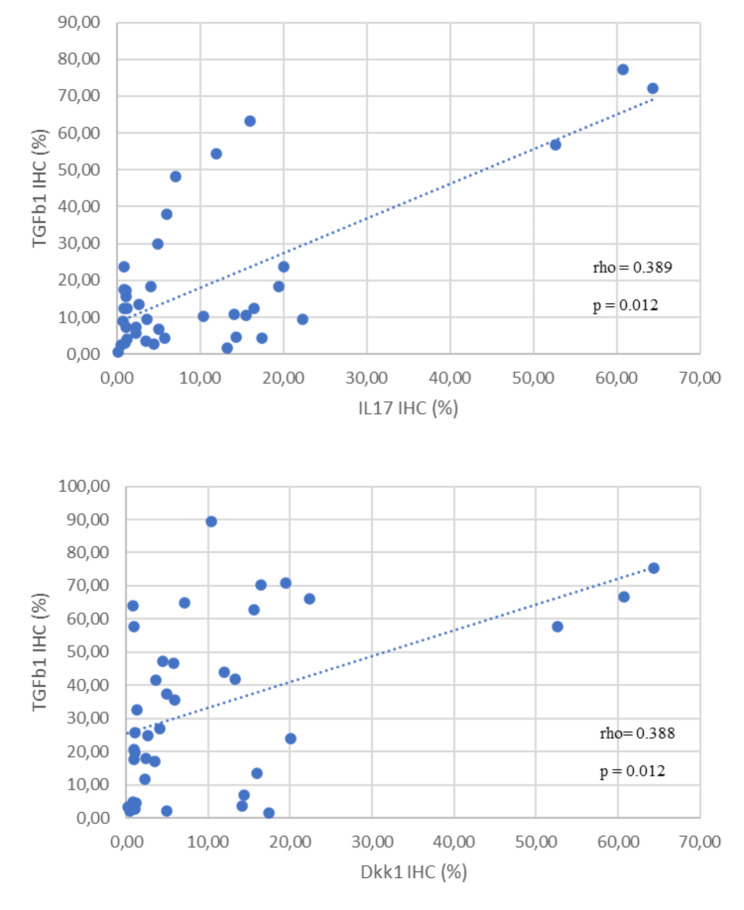
Correlation of IHC of TGF-β1 with IL-17 A, and Dkk1

The IHC expression for TGF-β1 was higher in PsA patients than in the other diseases (Table 4). The IHC reactivity for TGF-β1 in the synovial tissue (Fig. 4) was higher in patients with psoriatic arthritis (p 0.010) and was positively correlated with IL-17 A (r = 0.389, p = 0.012) and Dkk1 (r = 0.388, p = 0.012), (Fig. 5). Nevertheless, IL-17 A and Dkk1 IHC% were not statistically different among group diseases (p = 0.448 and p = 0.323, respectively), although the results were slightly higher for IL-17 A for PsA and AS (Table 4).

**Table 4:** IHC reactivity differences according to the diseases

	All N = 42	PsA N = 15	RA N = 8	OA N = 15	AS N = 4
BMP2	3.2 (0.8, 8.1)	3.6 (0.7, 26.4)	4.4 (1.5, 14.0)	1.7 (0.3, 8.1)	1.9 (0.8, 5.9)
BMP4	9.8 (5.9, 19.4)	7.7 (5.0, 16.9)	16.5 (6.6, 22.9)	9.6 (3.0, 20.5)	9.8 (6.6, 15.2)
DKK1	26.8 (6.8, 57.7)	23.8 (5.1, 62.2)	46.5 (17.6, 58.1)	20.8 (3.4, 62.8)	35.7 (15.6, 53.2)
Wnt5a	32.5 (22.9, 52.7)	31.5 (23.3, 57.8)	55.2 (42.7, 69.3)*	27.2 (22.2, 36.4)	22.9 (12.3, 57.4)
TGF-β1	4.9 (1.2, 14.3)	15.9 (7.5, 36.3)*	2.8 (1.0, 5.9)	2.4 (0.9, 10.4)	5.9 (2.7, 10.2)
IL-17 A	12.3 (4.6, 24.5)	17.2 (10.1, 59.9)	9.9 (3.4, 24.8)	9.4 (5.6, 15.7)	18.3 (2.9, 431)

Data as median (Q1-Q3). Kruskal-Wallis test.

^*^p < 0.05.

**Fig. 6:**
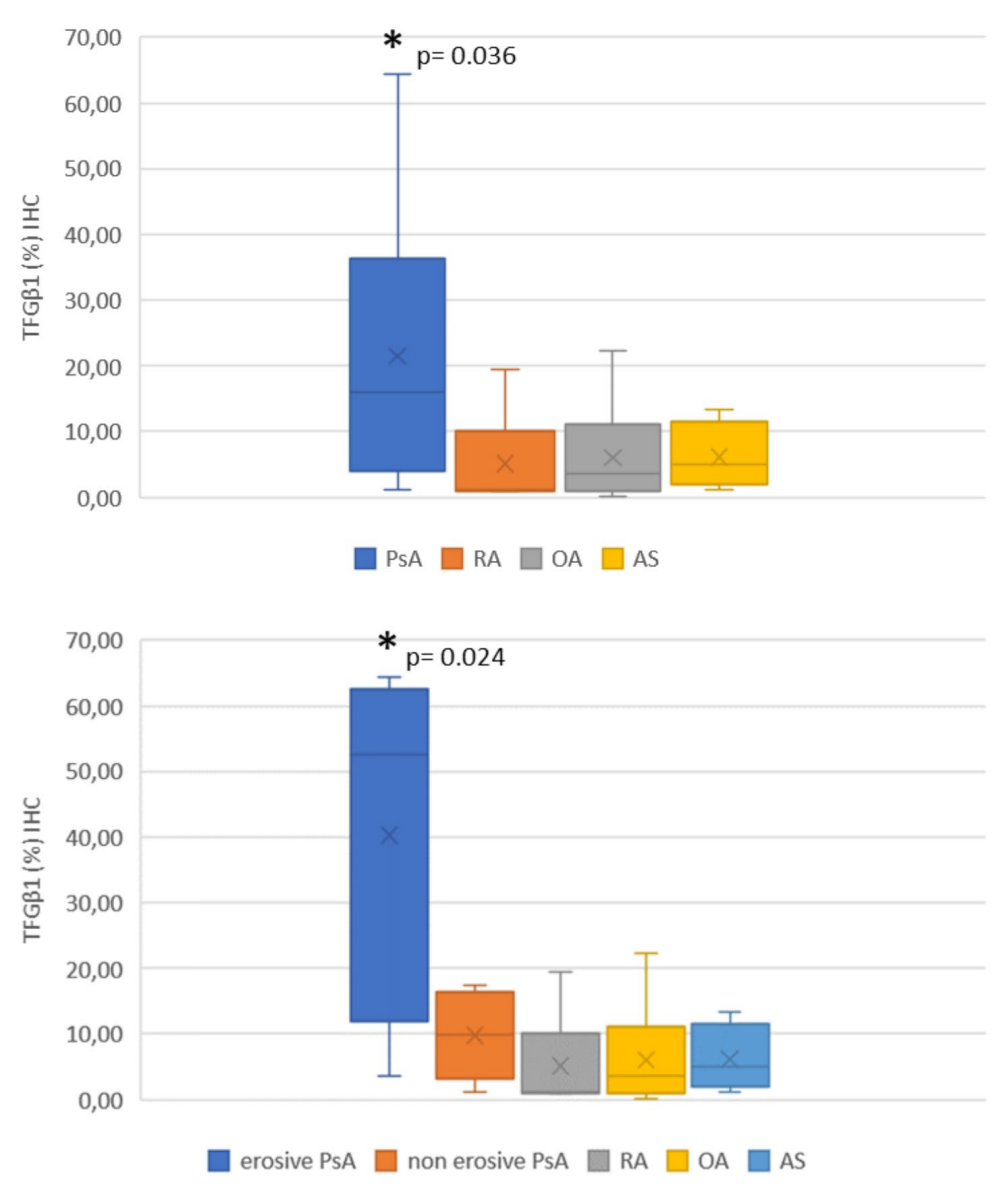
Differences in IHC of TFG-β1 between erosive and non-erosive diseases

When PsA patients’ group was divided between patients with (n = 6) or without (n = 9) radiologic erosive damage (Fig. 6), the TGF-β1 serum levels were significantly increased in PsA with erosions (p = 0.024). Following treatment with MTX, patients had lower IL-17 A gene expression in the synovial membrane than the other patients did (p < 0.0001), and in the regression logistic analysis (adjusted by age, gender, disease duration, and KL scale in the knee target), it had a correlation with erosive disease (p = 0.027).

The RA patients obtained higher serum concentrations of BMP2 than the other groups (Table 5). Additionally, Dkk1 in PsA, like in AS, obtained higher serum levels than in RA and OA; BMP4 and sclerostin were higher in OA; Wnt1, Wnt5b, TGF-β1, and IL-22 were higher in AS; the serum concentration of IL-6 was lower in OA, and higher levels of IL-17 A were seen in OA with respect to the other pathologies.

**Table 5:** Serum levels of proteins according to the diseases

Serum levels*	All n = 38	PsA n = 12	RA n = 7	OA n = 15	AS n = 4	p
DKK1 pgr/mL	3525 (1841–4791)	4215 (1894–5083)	2119 (791–3746)	2807 (1265–4762)	3826 (1909–5740)	0.648
Sclerostin ngr/mL	7.08 (5.23–10.99)	6.58 (5.79–9.95)	6.42 (2.90-12.32)	9.13 (5.10–8.63)	7.58 (5.18–8.63)	0.966
BMP2 pgr/mL	70.20 (57.57–99.29)	77.79 (61.10-96.65)	99.92 (57.87-103.05)	70.77 (64.43-107.82)	44.18 (43.03–44.18)	**0.043**
BMP4 pgr/mL	420 (287–550)	398 (264–548)	359 (244–532)	459 (241–620)	428 (321–526)	0.139
Wnt1 (ng/mL)	0.30 (0.21–0.47)	0.30 (0.24–0.54)	0.28 (0.18–0.58)	0.32 (0.17–0.53)	0.36 (0.23–0.46)	0.508
Wnt5a ngr/mL	55.11 (30.13–98.49)	54.84 (38.64–98.71)	37.37 (20.73–64.69)	56.93 (40.14–87.28)	68.67 (40.81–96.62)	0.767
TGFβ1 pgr/mL	1694 (1060–2038)	1551 (661–2232)	1530 (769–1871)	1529 (1015–1812)	2039 (1527–2372)	0.408
IL17A pg/mL	510 (347–941)	429.8 (121.9-672.1)	309.2 (241.8-463.4)	877.3 (499.4–1165)	486.2 116.8-516.7	0.492
IL22 ngr/mL	39.83 (17.72–7.22)	29.91 (21.76–92.93)	41.68 (11.83–60.80)	44.92 (16.69–65.89)	73.56 (47.40-103.91)	0.141
IL6 pgr/mL	63.72 (37.70-138.61)	87.41 (56.32-144.93)	99.56 (35.94-144.42)	34.95 (52.91–66.25)	92.21 (41.15-205.19)	0.397

*Data as median (Q1-Q3). Kruskal-Wallis test.

## Discussion

As the main source of IL-17 A, differentiated Th17 cells require TGF-β1 and IL-23 [17], and TGF-β1 is a pleiotropic cytokine that is involved in both suppressive and inflammatory immune responses [18]. TGF-β1 controls the proliferation, survival, activation, and differentiation of B cells as well as the development and functions of innate cells, including natural killer (NK) cells, macrophages, dendritic cells, and granulocytes. TGF-β1 is normally generated by macrophages during apoptotic cell clearance and contributes to buffering the inflammatory sequelae that are associated with phagocytosis, playing an important role in maintaining the tolerance by controlling survival, proliferation, and differentiation of the Th17 lymphocytes [19], and Th17 cells can be both immunoregulatory and pathogenic [20]. Our data showed that the immunohistochemical expression of TGF-β1 in the synovial tissue was higher in patients with PsA compared to those with RA, OA, and AS. Interestingly, patients with PsA who had developed bone erosions had the highest levels of TGF-β1 expression in IHC and these data could reflect a tissue healing effect.

In addition, in our patients, IL-17 A expression was also higher in PsA than it was in RA, and it was especially higher than it was in OA, and it seems to be like what is observed in AS, which shares pathogenic mechanisms with PsA. The generation of regulatory Th17 cells is promoted by the combination of TGF-β1 and IL-6 [21–23]. Pathogenic Th17 cells, however, require further stimulation with IL-23 [24, 25], although pathogenic Th17 cells can also be induced in cell culture without TGF-β1 and in the presence of IL-6, IL-1β, and IL-23. At low concentrations, TGF-β1 synergizes with IL-6 and IL-21 to promote IL-23 receptor expression and Th17-cell differentiation, whereas high TGF-β1 concentrations repress IL-23 receptor expression and promote Treg-cell differentiation [26]. Our findings show that patients with higher IL-23 gene expression were correlated with high expression levels of IL-17 A.

Since Dkk1 is an inhibitor of the Wnt pathway, which normally induces new bone formation, one might expect Dkk1 concentrations to be progressively lower along a spectrum of diseases that increase bone formation. However, consistent with most studies, the Dkk1 concentrations were seen as no different in patients with peripheral PsA compared to healthy controls and were higher in patients with AS compared to those with PsA [27, 28]. In our report, Dkk1 gene expression was higher in AS patients and correlated with IL17A, and serum levels were higher in PsA and AS but did not show statistical significance. Jadon et al. [29] found lower Dkk1 concentrations in patients with PsA with vertebral erosions and Li et al. [30] showed the combined action of TNFα and IL-17 A on hMSCs, which increased osteogenesis through the inhibition of Dkk1 and RANKL gene expression.

BMPs are considered cytokines that stimulate the formation of bone and cartilage, and they have been shown to play important roles in migration, proliferation, apoptosis, and differentiation of several cell types [31]. BMP2 induces bone and cartilage formation [32], and Osta et al. [33] saw that IL-17 decreased TNFα-induced BMP2 inhibition in vitro, with IL17A potentiating the effect of TNFα, and this fact may explain the ligaments ossification mechanisms as observed in AS. Our data show lower BMP2 and 4 gene expression in patients with PsA and higher IL-17 A synovial mRNA expression correlating with Dkk1. This fact is in accordance with the pathways that are implicated in new bone formation in SpA and include the IL-23/IL-17 axis and the BMP and Wnt signaling pathways [34]. Diarra et al. [35] were able, by inhibiting Dkk-1, to reverse the bone destructive pattern of a mouse model of rheumatoid arthritis to the bone-forming pattern of osteoarthritis. Decrease expression levels of the Wnt antagonists, Dkk1 and sclerostin, in SpA lead to increase Wnt signaling and osteoblast function, resulting in new bone formation. Mechanical stress also promotes inflammation and new bone formation in the entheses [34]. Moreover, Zhang et al. [36] showed that IL17 significantly inhibited osteoblasts differentiation induced by BMP2 in a murine model.

We found a higher serum level of IL-17 A in OA compared with PsA. In general, IL-17 A is prominently produced by the Th17 subtype of Th cells. These cells can act as either pathogenic or protective, depending on the cytokine milieu that stimulates them. Moreover, γδ T cells (another important source of IL-17) do not seem altered in the synovial tissue in patients with OA. Several studies suggested that serum IL-17 A is increased in patients with more severe OA (KL grade 2–4) with no correlation regarding levels in SF or synovium expression [37], and we have observed these high serum levels in older patients with severe knee OA.

Patients who were following treatment with MTX when the biopsy was performed had lower IL-17 A gene expression in the synovial membrane than the other patients, and it was correlated with erosive disease. It is known MTX dose-dependently suppresses the production of IL-17 A at the mRNA level by PBMCs from healthy donors and RA patients [38, 39].

Our study is limited due to its retrospective design and single-center approach and future longitudinal studies are needed to evaluate the real role that TGF-β1 plays in PsA with erosive damage in peripheral joints and its interaction with other cytokines involved in inflammation such as IL17A and Dkk-1. Moreover, the size of the patient’s samples is a bit small, but these studies provide estimates for biomarkers and effect sizes with respect to clinical outcomes, which are necessary to calculate the sample size and statistical power for future studies. Very small samples undermine the internal and external validity of a study. That is why these results should be considered with caution and this study should be replicated with larger sample size. The findings in the synovial membrane will be more enriching in PsA together with the study of peripheral entheses and techniques for obtaining enthesis tissue samples for study are currently being perfected [40, 41].

In summary, IL-17 A gene expression in the synovial membrane of patients with psoriatic arthritis is positively correlated with traditional osteo-destructive proteins and negatively correlated with the bone-forming proteins in peripheral arthritis. TGF-β1 (which is necessary for the activation of Th17 cells, but also involved in regeneration processes) immunoreactivity in synovial tissue, was higher in patients with erosive psoriatic arthritis in relation to the increased levels of IL-17 A and Dkk1 in the IHC.

## Data Availability

The datasets generated and/or analyzed during the current study are not publicly available due to privacy or ethical restrictions but are available from the corresponding author upon reasonable request.
